# Research Hotspots and Trends of Peripheral Nerve Injuries Based on Web of Science From 2017 to 2021: A Bibliometric Analysis

**DOI:** 10.3389/fneur.2022.872261

**Published:** 2022-05-20

**Authors:** Shiwen Zhang, Meiling Huang, Jincao Zhi, Shanhong Wu, Yan Wang, Fei Pei

**Affiliations:** ^1^Department of Rehabilitation Medicine and Physical Therapy, Graduate School, Heilongjiang University of Traditional Chinese Medicine, Harbin, China; ^2^Rehabilitation Center, The Second Affiliated Hospital of Heilongjiang University of Traditional Chinese Medicine, Heilongjiang University of Traditional Chinese Medicine, Harbin, China

**Keywords:** peripheral nerve injuries, CiteSpace, VOSviewer, bibliometric analysis, Web of Science

## Abstract

**Background:**

Peripheral nerve injury (PNI) is very common in clinical practice, which often reduces the quality of life of patients and imposes a serious medical burden on society. However, to date, there have been no bibliometric analyses of the PNI field from 2017 to 2021. This study aimed to provide a comprehensive overview of the current state of research and frontier trends in the field of PNI research from a bibliometric perspective.

**Methods:**

Articles and reviews on PNI from 2017 to 2021 were extracted from the Web of Science database. An online bibliometric platform, CiteSpace, and VOSviewer software were used to generate viewable views and perform co-occurrence analysis, co-citation analysis, and burst analysis. The quantitative indicators such as the number of publications, citation frequency, h-index, and impact factor of journals were analyzed by using the functions of “Create Citation Report” and “Journal Citation Reports” in Web of Science Database and Excel software.

**Results:**

A total of 4,993 papers was identified. The number of annual publications in the field remained high, with an average of more than 998 publications per year. The number of citations increased year by year, with a high number of 22,272 citations in 2021. The United States and China had significant influence in the field. Johns Hopkins University, USA had a leading position in this field. JESSEN KR and JOURNAL OF NEUROSCIENCE were the most influential authors and journals in the field, respectively. Meanwhile, we found that hot topics in the field of PNI focused on dorsal root ganglion (DRG) and satellite glial cells (SGCs) for neuropathic pain relief and on combining tissue engineering techniques and controlling the repair Schwann cell phenotype to promote nerve regeneration, which are not only the focus of research now but is also forecast to be of continued focus in the future.

**Conclusion:**

This is the first study to conduct a comprehensive bibliometric analysis of publications related to PNI from 2017 to 2021, whose bibliometric results can provide a reliable source for researchers to quickly understand key information in this field and identify potential research frontiers and hot directions.

## Introduction

Peripheral nerve injuries (PNI) are mainly caused by surgery and trauma and are common in clinical practice, with 13 to 23 per 1,00,000 people typically suffering from PNI (1–4). PNI is characterized by complex regeneration mechanisms, poor prognosis, and slow recovery (5–9), often leading to sensory and motor dysfunction and even lifelong disability, which not only seriously reduces the quality of life of patients but also brings a more serious health care burden to society (10–14). However, despite the increased understanding of the mechanisms of injury and regeneration, full functional recovery remains unsatisfactory in most patients (15, 16). With the rapid increase in the number of publications, it is becoming increasingly difficult for researchers to keep up with the latest findings (17, 18). It has been noted that researchers can benefit from an overview analysis of the domain knowledge structure and current hotspots (19). Therefore, bibliometric techniques are becoming increasingly popular as a quantitative analysis method by obtaining the above parameters (20). Currently, there is only one bibliometric analysis on PNI, with a review of publications that increased substantially during the First World War (21). Thus, a comprehensive bibliometric analysis of PNI is still very much lacking. In this study, a comprehensive bibliometric analysis was conducted in the field of PNI from 2017 to 2021, to help researchers quickly understand the knowledge structure and current hotspots in the field, to provide new ideas for developing new research topics, and to contribute to improving the quality of articles on PNI.

## Materials and Methods

### Data Acquisition and Search Strategy

The literature source was selected as the Science Citation Index Expanded (SCI-expanded) in Core Collection Indices of Web of Science (WoS). The literature search strategy was presented as follows: TOPIC: (peripheral nerve injury) OR TITLE: (peripheral nerve injury) OR TOPIC: (peripheral nerve injuries) OR TITLE: (peripheral nerve injuries). The time span was selected for the period from 2017 to 2021. The language was limited to “English”. The document type was “articles” and “review” and other types, such as early access, editorial material, meeting abstract, proceedings paper, book chapter, letter, correction, data paper, book review, were excluded. A total of 4,993 publications was retrieved. The last deadline was January 23, 2022.

### Data Extraction

Annual publication counts, citation frequency, country, institution, author, journal, keywords, and references were extracted from the data exported as “Full Record and Citation References”. The H-index and the average citations per item (ACI) for countries and institutions are extracted through the “Create Citation Report” function in the Web of Science database. Journal Impact Factor (JIF) and quartile categories Q1, Q2, Q3, and Q4 were extracted from the Journal Citation Reports in the Web of Science database.

### Data Analysis

Three bibliometric tools were used in this study. The online bibliometric platform was used in this study for country collaboration analysis and analysis of different country publications over the years (https://bibliometric.com/). CiteSpace, free software developed by Chen, is often used as a bibliometric tool to determine the structure and distribution of knowledge in a given field (22, 23). In our study, CiteSpace was used to perform collaborative analysis of institutions, co-citation relationships of authors, analysis of keywords, and analysis of references. It is worth noting that the nodes represent a type of project, such as country, institution, or author. The size and color of the node circles indicate the number or frequency of articles issued and the year of the appearance of these items, respectively, and the connecting lines between the nodes reflect the cooperation between different items (24). VOSviewer, another bibliometric software developed by Prof. van Eck and Waltman, with the ability to construct and view bibliometric maps, provides three types of maps, including network visualization maps, coverage visualization maps, and density visualization maps, each of which emphasizes a different aspect of the map, a detailed description of which can be found on the website (https://www.vosviewer.com/documentation) (25, 26). This study mainly applied the software for the author's co-authorship analysis and journal co-citation analysis.

## Results and Discussion

### Publication Outputs and Citation Trends

**Figure 1:**
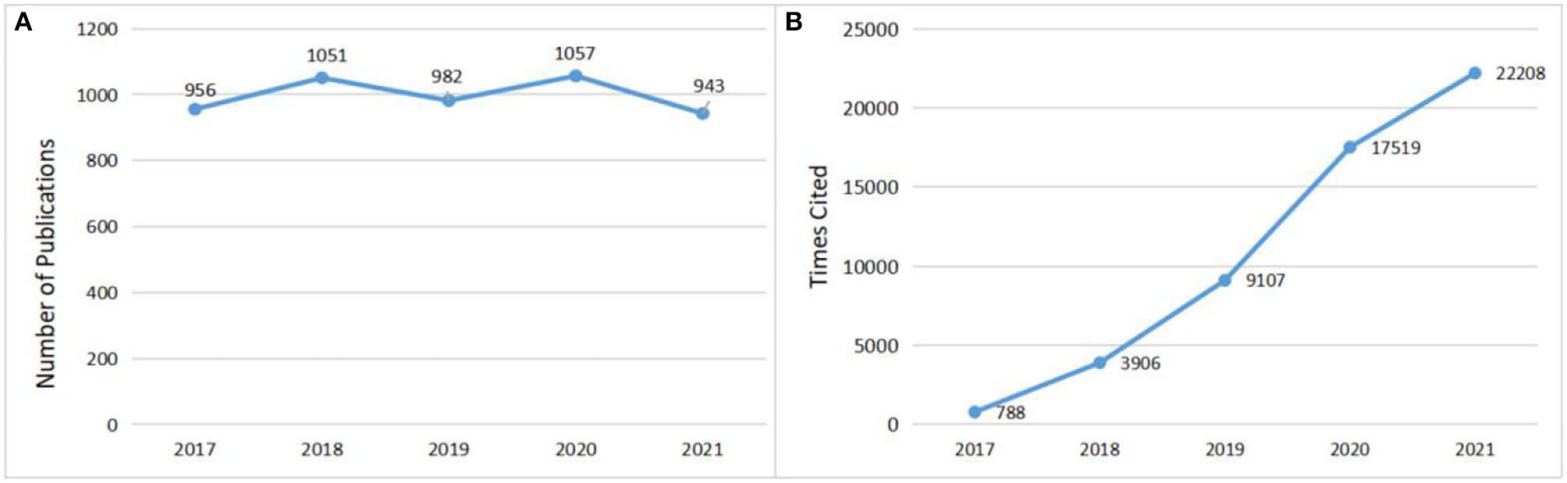
**(A)** The number of annual publications. **(B)** The annual times cited.

The annual publications and times cited can reflect directly the trends of scientific knowledge in a particular field. After the above literature screening, a total of 4,993 papers was included in the final analysis, of which 4,120 were original articles and 873 were reviews. The specific distribution of annual publications was shown in Figure 1A. As can be seen, despite small fluctuations, the number of annual publications had been high, with an average of more than 998 publications per year, indicating that PNI has been hot and has received continued attention from research scholars during the 5 years, and the field is expected to remain a research hotspot in 2022. In terms of citations, the cumulative total number of citations for these publications was 54,719 (43,332 after removal self-citations), with average citations per item of 10.92. The distribution of the number of citations per year can be seen in Figure 1B, with the number increasing year by year and a large increase in the number of citations between 19 and 20, with the highest number of citations in 2021 at 22,272. The heat of research on PNI remained high from 2017 to 2021 and attracted gradually the attention of annual publications and citations.

### Country

**Figure 2:**
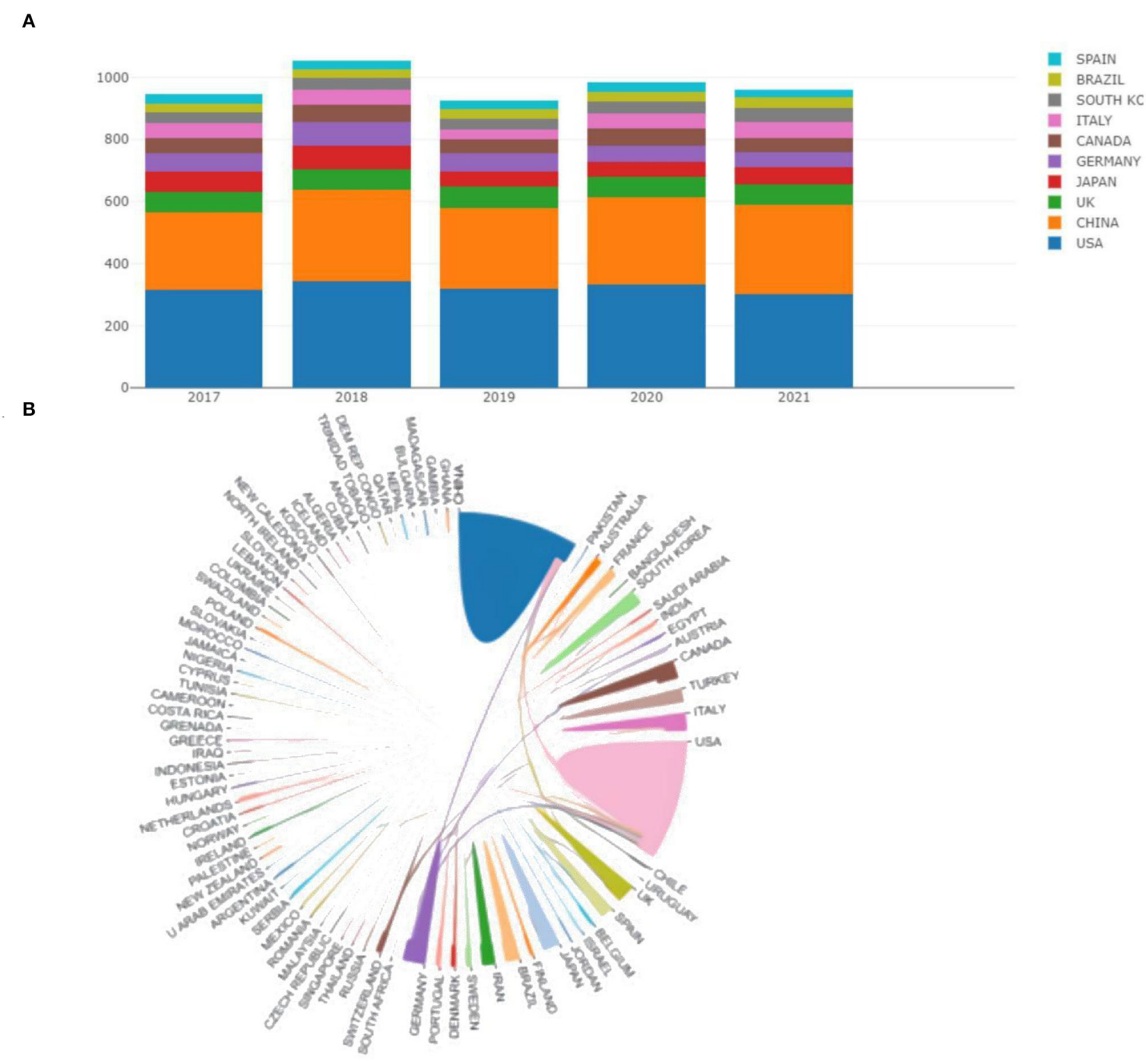
**(A)** The annual publication counts of the top 10 countries from 2017 to 2021. **(B)** Collaboration analysis among different countries.

The top 10 countries in terms of publications were presented in Figure 2A. According to the different color gradients, We can observe that the majority of the articles are from the United States and China. In specific, as shown in Table 1, we can understand that the US has the most publications in this field with 1,641 (32.87%), followed by China 1,307 (26.18%), UK 318 (6.37%), and Germany 302 (6.05%). The h-index, a new parameter for quantifying scientific achievement proposed by Jorge Hirsch, is defined as the number of papers with citation number ≥ h (27–29). It was evident from the Table that the USA and China still occupied the first and second positions in the h-index. ACI is another indicator to measure scientific contribution. The top 5 countries in ACI were England (17.56), Germany (15.19), the USA (14.8), Spain (13.52), and China (12.14). Overall, the US and China were significant influencers in all aspects including volume of publications, H-index, and ACI. Furthermore, as can be seen from Figure 2B, the cooperation between different countries was clearly shown. The thicker the line between the two countries indicates the more cooperative exchanges. It can be seen that the cooperation between the United States and China was closer.

**Table 1:** The top 10 most productive countries from 2017 to 2021.

**RANK**	**Country**	**Contribution**	**% of 4,993**	**h-index**	**ACI**
1	USA	1,641	32.87%	54	14.84
2	China	1,307	26.18%	44	12.2
3	England	318	6.37%	37	17.63
4	Germany	302	6.05%	33	15.24
5	Japan	297	5.95%	26	10.81
6	Canada	257	5.15%	28	13.37
7	Italy	234	4.69%	24	11.87
8	South Korea	195	3.91%	22	8.91
9	Brazil	159	3.18%	17	9.11
10	Spain	146	2.92%	23	13.56

### Institution

**Figure 3:**
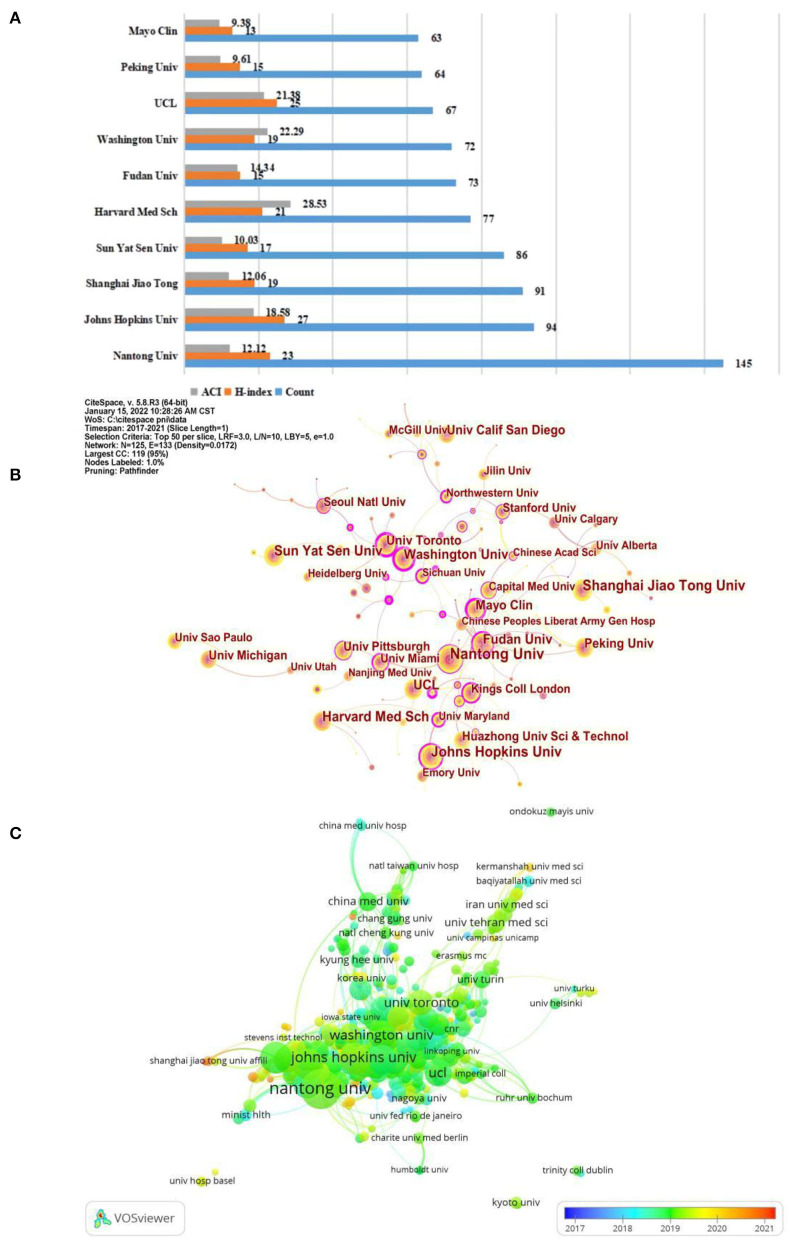
**(A)** The publication counts, h-index, and ACI of the top 10 most prolific institutions. **(B)** Visualization map of institutions' cooperative relationship by using CiteSpace software. **(C)** Coverage visualization map of institutions co-authorship analysis by using VOSviewer software.

The publication counts, h-index, and ACI of the top 10 most prolific institutions were shown in detail in Figure 3A. Among them, Nantong University in China ranked first with 148 articles, Johns Hopkins University in the United States ranked second with 117 papers, and Shanghai Jiao Tong University in China ranked third with 98 papers. In addition, Johns Hopkins also had the top H-index ranking at 27 and the ACI (18.7) ranking at Johns Hopkins was also in the higher position. As such, Johns Hopkins University had a leading position in this field. This study also conducted an institutional cooperation analysis using CiteSpace software (Figure 3B). The betweenness centrality (BC) of a node in a network reflects the importance of the node's position in the network, and BC is an indicator of the centrality of a node (30–32). In general, nodes with BC values of more than 0.1 have a purple outer ring, which also indicates that the node occupies a key position connecting a large number of nodes. Of all these institutions, 36 institutions had BC values above 0.1. The top five institutions with the highest BC values were, Rutgers State Univ (0.66), Mayo Clin (0.6), Ohio State Univ (0.54), Washington Univ (0.46), Univ Toronto (0.42). It can be seen that most of these institutions were from the United States, again indicating the influence of the United States in the field. In parallel to the CiteSpace software, we also conducted a collaborative institutional analysis of organizations through VOSviewer. As displayed in the overlay visualization in Figure 3C, the nodes representing institutions were marked with different colors depending on the average year of appearance (AAY) of each institution, where institutions with relatively early average years of appearance were closer to blue or dark blue, e.g., Gunma University, University of Strasbourg, Kitasato University, in contrast, many institutions marked in red or dark red may be relatively new participants in the field, e.g., Queens University, Chinese People's Liberation Army General Hospital, Ewha Womans University, etc.

### Co-authorship and Co-citation Author

**Figure 4:**
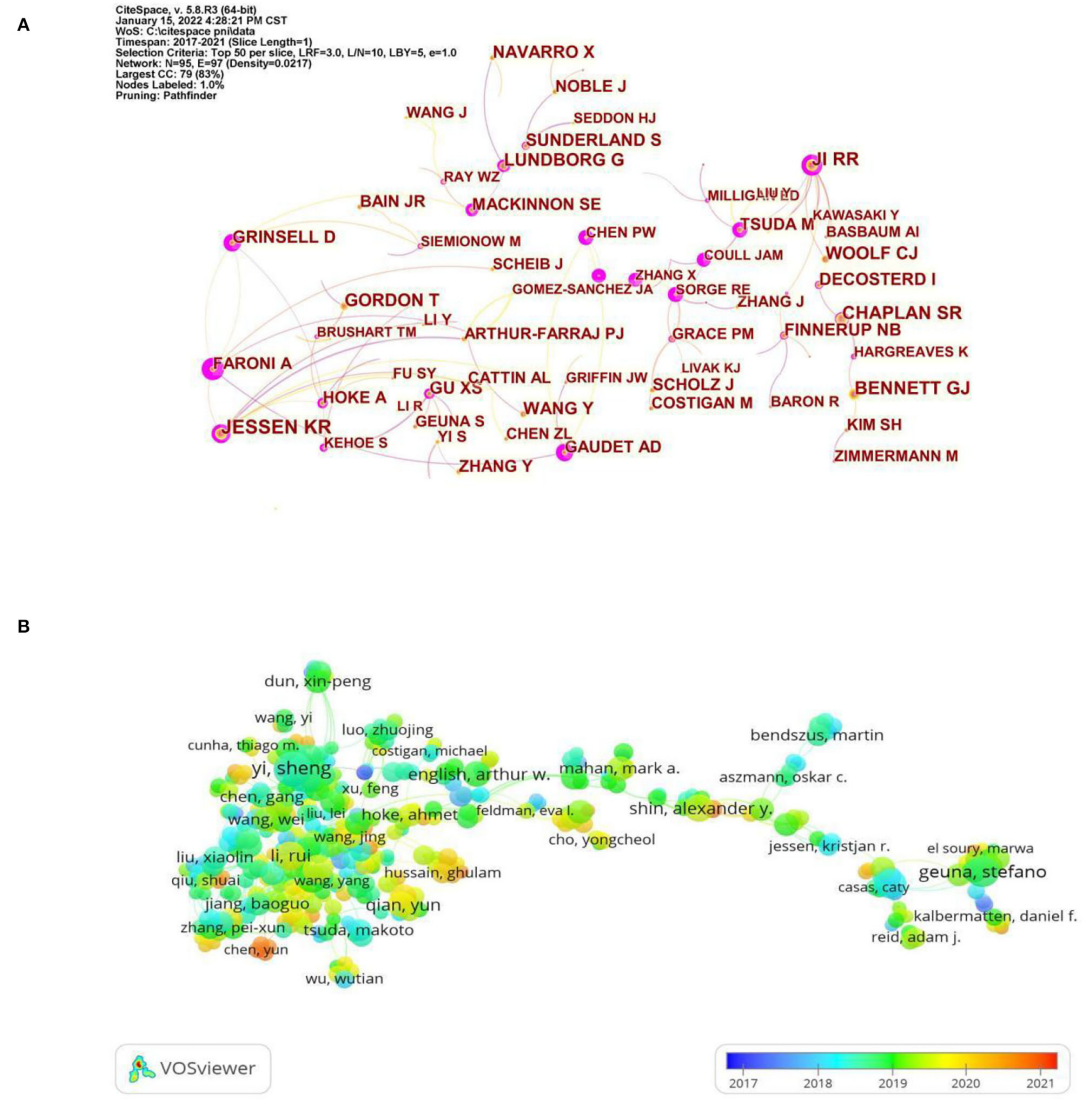
**(A)** Visualization map of author co-citation analysis by using CiteSpace software. **(B)** Coverage visualization map of author co-authorship analysis by using VOSviewer software.

The analysis co-citation author is often used to reveal the key authors in the co-citation network of a particular field. The more citations an author receives, the more influence he or she has. The top five most-cited authors were, JESSEN KR (446), CHAPLAIN SR (335), JI RR (332), BENNETT GJ (328), and GORDON T (289). JESSEN KR is with the Department of Cell and Developmental Biology, University College London, where he focuses on Schwann cells as a fulcrum to explore the underlying mechanisms of peripheral nerve regeneration (33–36). As can be seen from Figure 4A, JESSEN KR collaborated closely with Arthur-Farraj P at the Department of Cell and Developmental Biology, University College London, and they had published more than 10 articles together. Of these, an article published in NEURON titled “c-Jun Reprograms Schwann Cells of Injured Nerves to Generate a Repair Cell Essential for Regeneration”, which was cited 431 times for the study, found that the transcription factor c-Jun in Schwann cells can play a role in determining the expression of trophic factors and adhesion molecules, regenerative track formation, and myelin clearance, controlling the unique regenerative potential of peripheral nerves by activating repair programs in Schwann cells and creating cells that support regeneration, suggesting that a single glial transcription factor c-Jun is critical for the repair of damaged nerves (37). From the above results, it can be seen that JESSEN KR was one of the most influential authors in the field due to his high citation frequency and close communication with other authors in the field. The analysis of the authors' co-authorship relationships is beneficial in revealing the current collaborations within the field. In Figure 4B, the VOSviewer software generated a superimposed visualization of the author's co-authorship analysis. It can be observed that the group with Yi Sheng as the core was large, and there was more cooperation within the group. Yi Sheng worked at Nantong University, China, where he focused on microRNA (38, 39), and Schwann cells (40, 41). Notably, most of the authors in this group were from East Asia, and there was less collaboration with authors from Europe. In the future, authors in this field should strengthen cooperation and exchange to mutually improve their international influence. Following the color gradient shown in the bottom right corner, we can find that the authors represented by Chen Yun were relatively young researchers in the field.

### Journal

**Figure 5:**
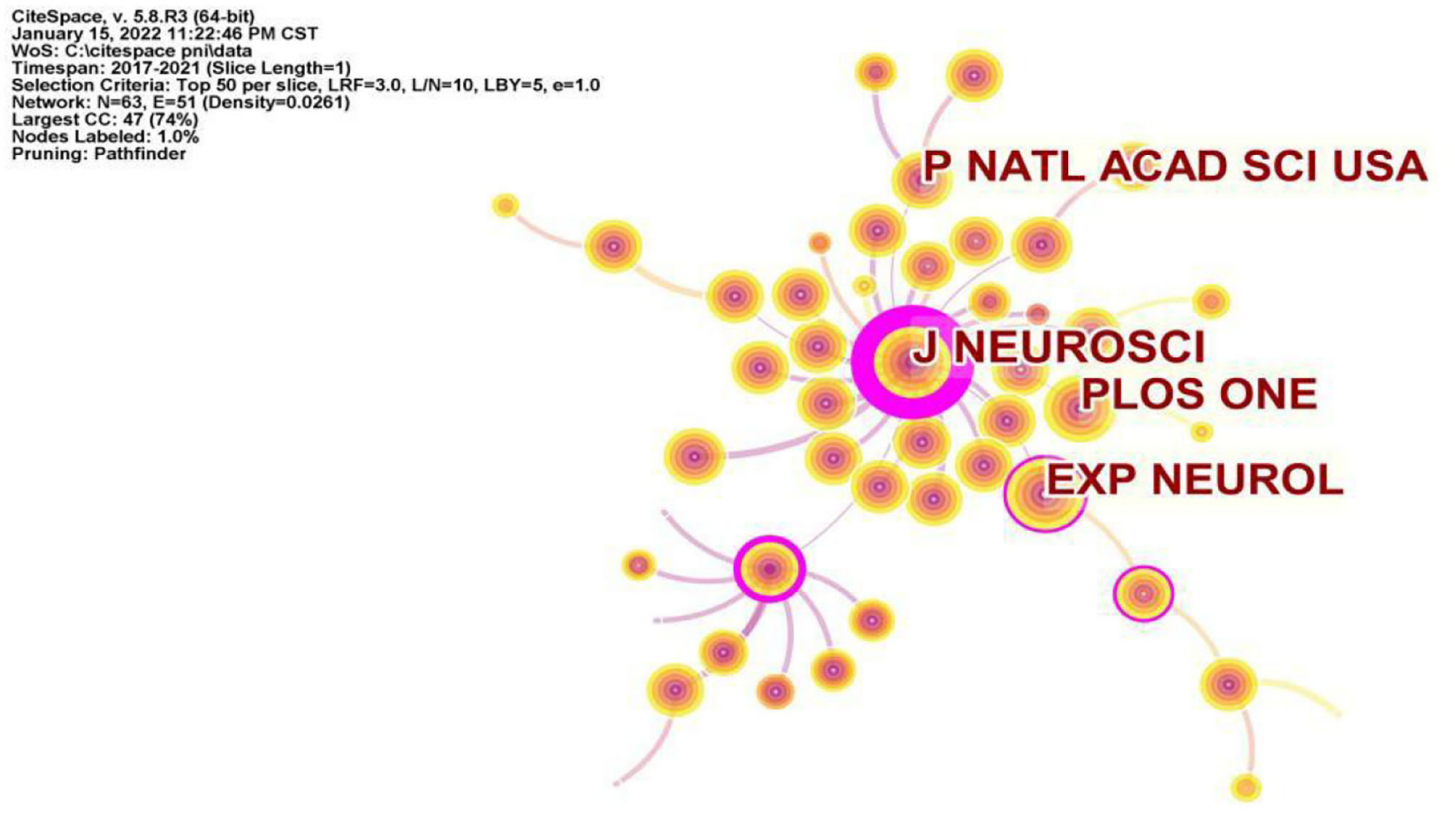
Visualization map of journal co-citation analysis by using CiteSpace software.

In total, more than 1,000 journals appeared in this research area, according to statistics. The top 10 active journals published 817 papers on PNI, accounting for 16.36% of all 4,993 papers. Among them, as shown in Table 2, NEURAL REGENERATION RESEARCH (156 articles) published the most articles, SCIENTIFIC REPORTS (99 articles) ranked second, followed by PAIN (92 articles) and INTERNATIONAL JOURNAL OF MOLECULAR SCIENCES (87 articles). In particular, the JIF of a journal is an important factor parameter in evaluating its value and the value of the publications it includes. Among the top 10 academic journals, JOURNAL OF NEUROSCIENCE had the highest JIF and was classified as Q1, which focused on neurophysiology (42, 43), neurodevelopment (44, 45), and brain imaging (46, 47). Interestingly, among the top 10 academic journals, 7 were in the neuroscience category, 1 called SCIENTIFIC REPORTS was a general journal, and the other 3, PAIN, INTERNATIONAL JOURNAL OF MOLECULAR SCIENCES, and MOLECULAR PAIN focused on the direction of pain and molecular biology. In addition to the number of publications, the impact of journals depends on the number of times they are co-cited in a particular field of study. For this study, CiteSpace software was used for co-citation journal analysis. As can be seen in Figure 5, the most cited journals were JOURNAL OF NEUROSCIENCE [2,681], followed by

**Table 2:** The top 10 most productive journals from 2017 to 2021.

**No**.	**Journal**	**Output**	**% of**	**JIF**	**Quartile in**
			**4,993**	**(2020)**	**category**
					**(2020)**
1	Neural regeneration research	156	3.13%	5.135	Q2
2	Scientific reports	99	1.98%	4.38	Q1
3	Pain	92	1.84%	5.135	Q2
4	International journal of molecular	87	1.74%	5.924	Q1/Q2
	Sciences				
5	Molecular pain	68	1.36%	3.395	Q3
6	Experimental neurology	60	1.20%	5.33	Q2
7	Frontiers in cellular neuroscience	55	1.10%	5.505	Q1
8	Neuroscience	54	1.08%	3.59	Q3
9	Journal of neuroscience	50	1%	6.167	Q1
10	Neuroscience letters	50	1%	3.046	Q3

EXPERIMENTAL NEUROLOGY (2,226), PLOS ONE (2,168), and P NATL ACAD SCI USA (1,898). In summary, JOURNAL OF NEUROSCIENCE was the most influential journal in this field.

### Analysis of Keywords Co-citation, Clustering, Timeline, and Burst

**Figure 6:**
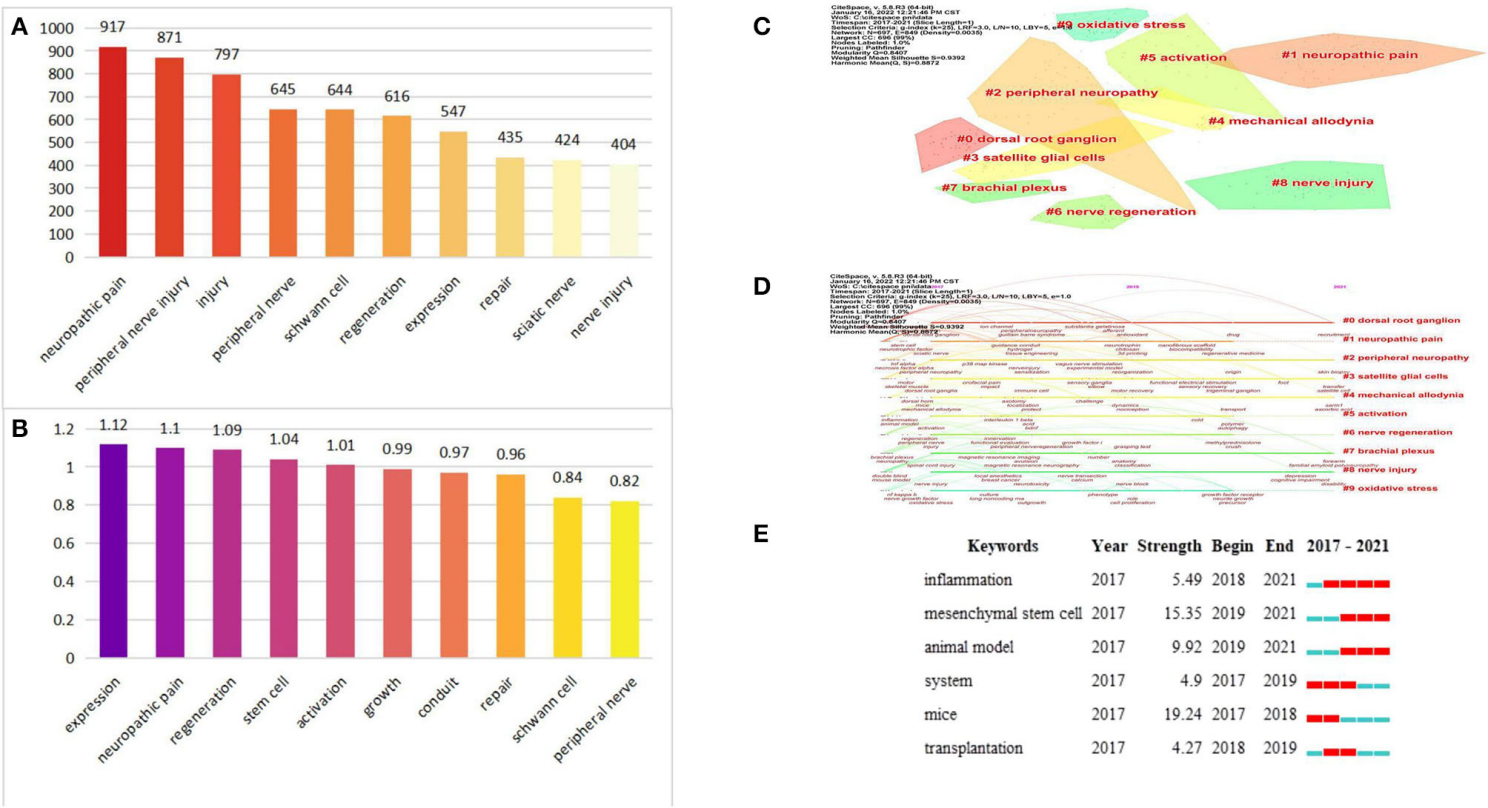
**(A)** The top 10 high-frequency keywords. **(B)** The top 10 high BC value keywords. **(C)** The cluster view map by using CiteSpace software. **(D)** The timeline view map by using CiteSpace software. **(E)** Top 6 Keywords with the Strongest Citation Bursts.

For bibliometrics, a useful method used to identify research hotspots is keyword co-occurrence analysis (48). Figures 6A,B showed the top 10 high-frequency keywords and high BC value keywords. After excluding the keywords “peripheral nerve”, “peripheral nerve injury”, “nerve injury”, and “injury”, other keywords “neuropathic pain”, “Schwann cell”, “regeneration”, “expression”, “sciatic nerve”, “stem cell”, “activation”, “growth”, and “conduit” were related to the study of mechanisms of peripheral nerve repair and regeneration and improvement of neuropathic pain. It should be noted that the modularity value (Q value) and the mean silhouette value (S value) are two important parameters to evaluate the effect of mapping. Q > 0.3 and S > 0.7 indicate significantly clustered (49, 50). In this present study, the Q value was 0.8407 and the mean S value was 0.9392, indicating that these clusters were effective and had good homogeneity. As shown in Figure 6C and Table 3, the top five clusters were, “dorsal root ganglion”(DRG) (#0), “neuropathic pain” (#1), “peripheral neuropathy” (#2), “satellite glial cells” (SGCs) (#3), and “mechanical allodynia” (#4). Meanwhile, we also provided a timeline view of the main clusters in Figure 6D, which can be used to understand the evolutionary characteristics of each cluster according to the timeline. It can be seen that “DRG” (#0), “peripheral neuropathy” (#2), “SGCs” (#3), “mechanical allodynia” (#4), “brachial plexus” (#7), and “nerve injury” (#8) were still the focus of research in 2021. Notably, neuropathic pain is one of the chronic pains which is often triggered by PNI and caused by damage to the somatosensory nervous system, with common clinical features such as mechanical allodynia. Neuropathic pain is challenging to cure, and there is currently no known cure (51, 52). DRG is widely recognized as a potential target for the treatment of chronic pain due to its ability to transmit harmful stimulus information to the cerebral cortex (53, 54). SGCs, a type of cell surrounding sensory neurons in the DRG, can communicate with sensory neurons through gap junctions and chemical signals to regulate chronic pain when activated after PNI (55). Recently, a new study revealed that DRG-SGCs show stem cell properties and can be effectively differentiated into sensory neurons, providing a basis for clinical treatment of chronic pain, suggesting that the potential of satellite glial cells is enormous (56). Mechanical allodynia, DRG and SGCs are some of the other hot keywords mentioned above, which means that exploring the pathophysiological mechanisms of neuropathic pain is a hot research topic. Keywords bursts refer to those that have been frequently cited within one period of time. From Figure 6E, we can find that blue represents 2017–2021, the left end of red indicates the start time of emergence, and the right end time of emergence, “inflammation”, “MSCs”, and “animal model” are important research directions and will continue to be focused on in the future. In addition, higher burst values indicate that they have received special attention and represent, to some extent, the research frontier in the field of study during the corresponding time interval. From the perspective of burst values, we can observe that the strongest burst strength among the three terms is MSCs. MSCs, a type of pluripotent stem cells from the mesoderm, can differentiate into neural cells to regulate the microenvironment of the injury zone. Currently, MSCs are often used in combination with tissue engineering, and it has been shown that nerve conduits loaded with bone marrow MSCs can both promote nerve growth and regulate the microenvironment of the injured area thereby effectively improving the regeneration and repair of PNI, which may be the reason why MSCs have been focused on in recent years, but so far, the differentiation mechanism and the ability of MSCs to secrete neurotransmitters are still unclear, which is an urgent issue to be addressed before MSC therapy can be applied clinically (57).

**Table 3:** Cluster analysis of keywords.

**ID**	**Size**	**Sihouette**	**Year**	**Lable**
0	50	0.909	2017	dorsal root ganglion
1	48	0.896	2017	neuropathic pain
2	38	0.934	2017	peripheral neuropathy
3	38	0.948	2018	satelite glial cells
4	33	0.968	2017	mechanical allodynia
5	31	0.94	2017	activation
6	31	1	2017	nerve regeneration
7	30	0.979	2017	brachial plexus
8	30	0.951	2018	nerve injury
9	30	0.964	2018	oxidative stress

### Analysis of References Co-citation, and Burst

**Figure 7:**
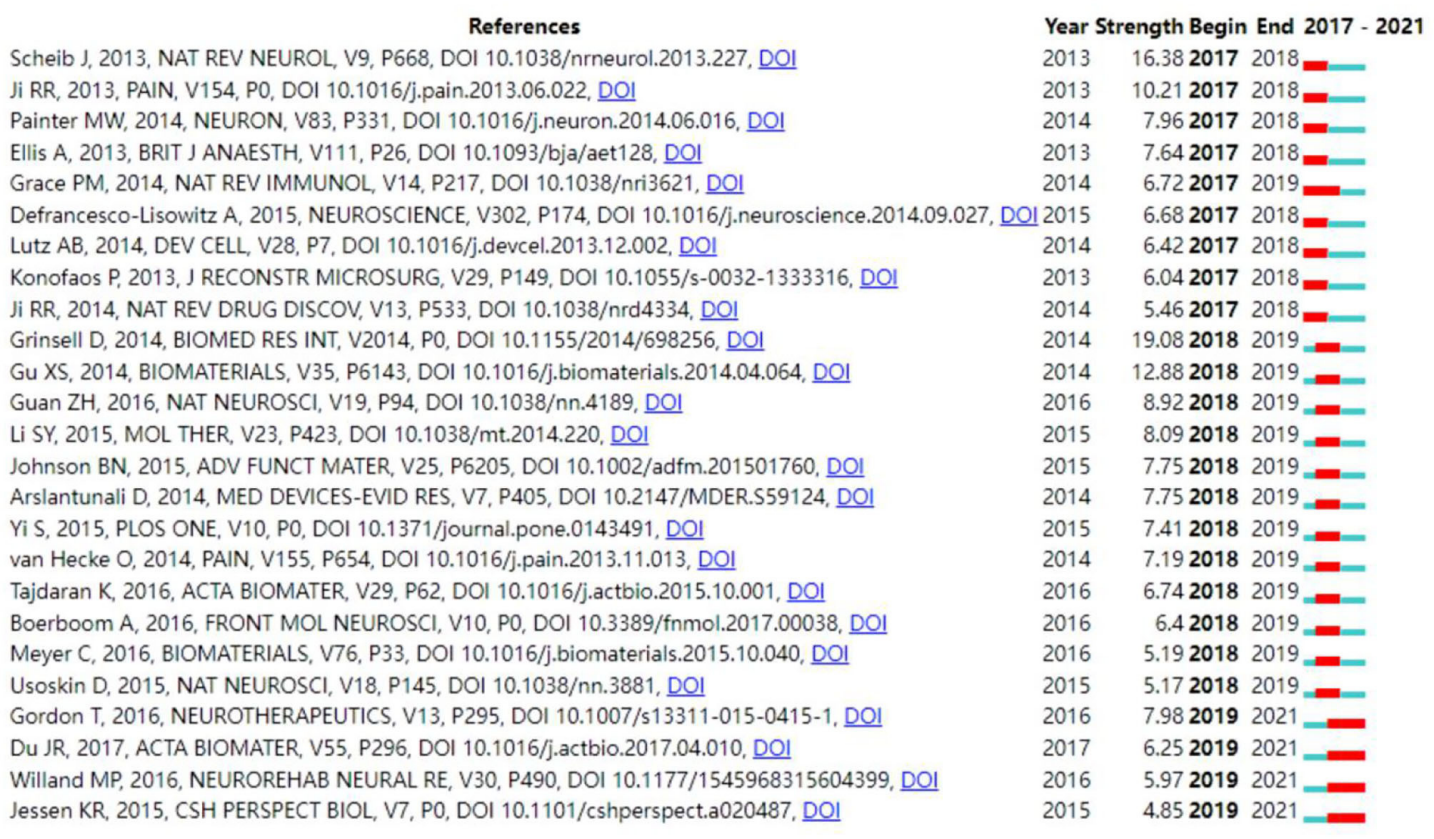
TOP 25 References with the strongest citation bursts.

The analysis of references is a significant factor in bibliometric studies (58). The top 10 most cited references were shown in Table 4, from which it can be found that the article written by Jessen KR has the highest number of co-citations, 229, and the article written by Jessen KR appears twice, indicating that Jessen KR has a certain social influence in this research area, which is consistent with the findings above. Furthermore, three articles with Schwann cells as a topic appeared in the top ten highly cited literature, indicating the key position of Schwann cells in the field of PNI. Figure 6C showed the top 25 references with the strongest citation bursts, which can be observed mainly in terms of both burst value and burst time. One of the references with the strongest burst values was written by Scheib et al. (59). In this study, they found that the rodent model of nerve injury, while adding to our understanding of peripheral nerve regeneration, does not adequately recapitulate the human situation, as human axons usually need to extend longer distances than mice after PNI, and due to the lack of proximal neuronal contact over time, distal hemopoietic cells and target tissues may atrophy and thus impede axon extension, and then suggested that the rodent model of chronic denervation can recapitulate an environment similar to that of human nerve injury, which would be more useful for the study of human nerve injury regeneration methods. In terms of burst timing, Figure 7 showed that most of the reference bursts have ended, but some continue into 2021, mainly related to the themes of “a three-dimensional hierarchically aligned fibrin nanofiber hydrogel (AFG)” (60), “brief electric stimulation” (61, 62), and “Schwann cells” (63). Among them, AFG, prepared by electrospinning and molecular self-assembly, with the characteristics of hierarchically aligned topography and low elasticity, is utilized in the form of a combination of tissue engineering therapeutic techniques to build an effective microenvironment and facilitate the role of Schwann cells in promoting peripheral nerve regeneration (60). Combined with the burst keyword analysis, we can observe that the combination with tissue engineering technology to improve the functional recovery rate of PNI has now become a key topic of interest and is expected to be followed up continuously in the future. In addition, it is worth mentioning that whether from high-frequency keywords, highly cited literature, or burst citation literature we can find that the study of the physiopathological mechanism of PNI based on Schwann cell has been the focus of attention of related scholars, showing great potential to accelerate peripheral nerve regeneration. Jessen KR et al. (63, 64) reviewed the development of Schwann cells and their central role in reprogramming the repair of post-injury nerves, which provides a solid basis for the development of more effective therapeutic approaches. Schwann cells are glial cells in the peripheral nervous system that are reprogrammed into a series of specific repair phenotypes to promote nerve regeneration after nerve injury (10, 65). However, maintenance of the repair phenotype is often brief and unstable, which contributes to the undesirable neural regeneration outcome in humans. Therefore, the study of promoting and maintaining the repair Schwann cell phenotype is of great relevance for promoting peripheral nerve regeneration, which is one of the important reasons why Schwann cells are a hot topic. Currently, some strategies based on promoting and maintaining the repair Schwann cell phenotype have been revealed, including low-intensity ultrasound (66), histone deacetylases (67), superparamagnetic iron oxide nanoparticles mediated magnetic actuation (68), etc. However, although the exploration of more effective treatment strategies is indeed the focus of current research, as more innovative treatments increasingly emerge, we believe that optimizing treatment strategies and conducting clinical evaluation practices to translate them into reality as early as possible are more critical issues for future researchers to address.

**Table 4:** The top 10 most cited references.

**No**.	**Title**	**Author**	**count**
1	The repair Schwann cell and its function in regenerating nerves	Jessen KR	229
2	Peripheral nerve regeneration: experimental strategies and future perspectives	Alessandro Faroni	116
3	Macrophage-Induced Blood Vessels Guide Schwann Cell-Mediated Regeneration of Peripheral Nerves	Anne-Laure Cattin	114
4	Pain regulation by non-neuronal cells and inflammation	Ru-Rong Ji	109
5	Peripheral nerve reconstruction after injury: a review of clinical and experimental therapies	D Grinsell	107
6	Neuropathic pain	Luana Colloca	105
7	Pharmacotherapy for neuropathic pain in adults: a systematic review and meta-analysis	Nanna B Finnerup	101
8	Role of macrophages in Wallerian degeneration and axonal regeneration after peripheral nerve injury	Peiwen Chen	94
9	Different immune cells mediate mechanical pain hypersensitivity in male and female mice	Robert E Sorge	88
10	Schwann Cells: Development and Role in Nerve Repair	Jessen KR	88

## Limitation

First, only a single database, Web of Science Core Collection, was selected for analysis in this study, and other large medical databases such as PubMed, Scopus, and EMBASE were excluded and this study was limited to, language type, file type, and time span, which may lead to underrepresentation of potential research in this field and weaken its impact. In addition, some of the most recently published influential papers may not appear in our study due to the constant updating of databases. However, the amount of data collected in this study was large enough and adequate to reflect the current state of research in the field of PNI.

## Conclusion

This is the first study to conduct a comprehensive bibliometric analysis of publications related to PNI from 2017 to 2021. Our findings indicated that the number of annual publications in the field of PNI remains high, with an average of more than 998 publications per year and an increasing number of citations yearly, up to 22,272 citations in 2021. Meanwhile, we found that hot topics in the field of PNI focused on DRG and SGCs for neuropathic pain relief and on combining tissue engineering techniques and controlling the repair Schwann cell phenotype to promote nerve regeneration, which are not only the focus of research now but will continue to be in the future. In summary, this bibliometric analysis will help researchers to quickly understand the knowledge structure and current hotspots in the field, provide new directions and ideas for research topics, and contribute to higher quality articles in the peripheral nerve field.

## Data Availability Statement

The original contributions presented in the study are included in the article/supplementary material, further inquiries can be directed to the corresponding authors.

## Author Contributions

These articles were retrieved and downloaded by SZ, MH, and JZ. Data were extracted from each article by SZ and SW. SZ, MH, JZ, and SW analyzed the data and wrote the manuscript. YW and FP were responsible for overseeing the project, revising drafts, and approving the final manuscript. All authors contributed significantly to the final article.

## Funding

This research was supported by the National Natural Science Foundation of China (81973926), the Natural Science Foundation of Heilongjiang Province (LH2021H090), and the Heilongjiang University of Chinese Medicine Outstanding Innovative Talents Support Program research project (2018RCL10).

## Conflict of Interest

The authors declare that the research was conducted in the absence of any commercial or financial relationships that could be construed as a potential conflict of interest.

## Publisher's Note

All claims expressed in this article are solely those of the authors and do not necessarily represent those of their affiliated organizations, or those of the publisher, the editors and the reviewers. Any product that may be evaluated in this article, or claim that may be made by its manufacturer, is not guaranteed or endorsed by the publisher.
